# GCIP functions as a tumor suppressor in non-small cell lung cancer by suppressing Id1-mediated tumor promotion

**DOI:** 10.18632/oncotarget.2075

**Published:** 2014-06-07

**Authors:** Kuan-yu Chen, Chao-chung Chen, Yau-lin Tseng, Yi-chien Chang, Ming-Chung Chang

**Affiliations:** ^1^ Institute of Biotechnology, National Cheng Kung University, Tainan, Taiwan; ^2^ Department of Biotechnology, College of Medicine and Nursing, Hung Kuang University, Taichung, Tainan; ^3^ Department of Nutrition, College of Medicine and Nursing, Hung Kuang University, Taichung, Tainan; ^4^ Department of Surgery, National Cheng Kung University Medical College and Hospital, Tainan, Taiwan

**Keywords:** GCIP, NSCLC, Id1, tumor suppressor

## Abstract

Grap2 and cyclin D1 interacting protein (GCIP) has been recognized as a putative tumor suppressor, but the molecular mechanisms underlying its anti-tumor properties remain undefined. Here, we report that GCIP is frequently downregulated in non-small cell lung cancer (NSCLC) tissues. Binding assays indicated that inhibitor of DNA binding/differentiation 1 (Id1) interacts with GCIP in the nucleus. Ectopic GCIP expression in the highly invasive NSCLC cell line, H1299, inhibited proliferation, colony formation, invasion and migration, and increased susceptibility to anticancer drugs. Conversely, silencing GCIP expression in the minimally invasive NSCLS cell line, A549, increased proliferation, colony formation, invasion, and migration in vitro, and increased survival and resistance to anticancer drugs. GCIP also suppresses tumorigenicity of NSCLC cells in vivo and GCIP suppresses NSCLC progression is mediated in part by interfering with Id1 signaling, which was confirmed in conditionally induced stable cell lines. In addition, GCIP downregulates the expression of Id1, and GCIP and Id1 are inversely expressed in NSCLC cell lines and specimens. Taken together, these results suggest that GCIP is a potential tumor suppressor in NSCLC and that suppression of Id1-mediated oncogenic properties may be a key mechanism by which GCIP can potently suppress NSCLC tumor progression.

## INTRODUCTION

Lung cancer is a leading cause of cancer death worldwide. More than 80% cases are classified as non-small cell lung cancer (NSCLC), which is characterized by its poor prognosis and resistance to antineoplastic drugs both *in vitro* and *in vivo* [1, 2]. Patients with NSCLC have a 5-year survival rate of less than 15% [3]. To improve the survival of patients with NSCLC, it is important to elucidate the signaling pathways regulating both NSCLC tumor promotion and suppression to identify novel prognostic markers and potential therapeutic targets[4, 5].

The inhibitor of DNA binding/differentiation (Id) proteins belong to the dominant-negative helix-loop-helix (dnHLH) family of proteins, which lack a basic domain for DNA binding [6]. Among four types of Id proteins (Id1, Id2, Id3, and Id4), Id1 has been extensively studied in various cancers and is linked to tumorigenesis, as aberrant elevation of Id1 has been found in over 20 types of human cancer [7]. Moreover, high Id1 expression levels are correlated with aggressive and high-grade cancer, as well as poor clinical outcome in different tumor types [8-13]. In addition, among the identified genes that mediated breast cancer metastasis to the lungs, Id1 was identified as one of the most active [14]. Furthermore, Id1 is a novel prognostic factor in NSCLC patients [15] and is a common mediator of NSCLC progression and metastasis in both smokers and nonsmokers [16].

GCIP is a 40-kDa HLH leucine zipper protein, which is also recognized as a dnHLH protein [17] and was originally identified to be a human Grap2 and cyclin D-interacting protein [18] and also was recognized as a human homologue of the MAID protein (HHM) [19] and a D-type cyclin-interacting protein 1 (DIP1) [20]. Although several proteins, such as nuclear p29, Rad associated with diabetes (Rad), ribosomal phosphoprotein (P0), and oligodendrocyte transcription factor 1 (Olig 1), interact with GCIP [21-25], its physiological function remains largely undefined. Previous studies indicated that GCIP is expressed mainly in terminally differentiated tissues and might play an important role in controlling cell differentiation and proliferation [26]. In addition, overexpression of GCIP in mouse liver suppressed diethylnitrosamine (DEN)-induced liver tumors in transgenic mice [27], and mice lacking GCIP (Maid) expression in the liver are prone to earlier development of hepatocellular carcinomas (HCCs) and hepatocellular adenomas (HCAs) [28]. In addition, expression of GCIP was reduced in several cancer tissues with tumor progression and metastasis, including breast, prostate and colon tumor tissues [22]. Furthermore, decreased expression of GCIP correlates with poor patient prognosis in breast cancer [29].

Although GCIP is considered a putative tumor suppressor in breast, colon and liver cancers, its role in NSCLC tumor progression remains unknown. In this report, we evaluated the expression of GCIP in NSCLC and explored its role in NSCLC progression. Our results revealed that GCIP expression was significantly downregulated in NSCLC tissues, and the antitumor activity of GCIP was mechanistically mediated by the ability of GCIP not only to interact with Id1 but also to suppress its transcriptional activity, and thereby, increasing the susceptibility to chemotherapeutic agents.

## RESULTS

### GCIP expression is significantly downregulated in invasive NSCLC tissues

**Figure 1:**
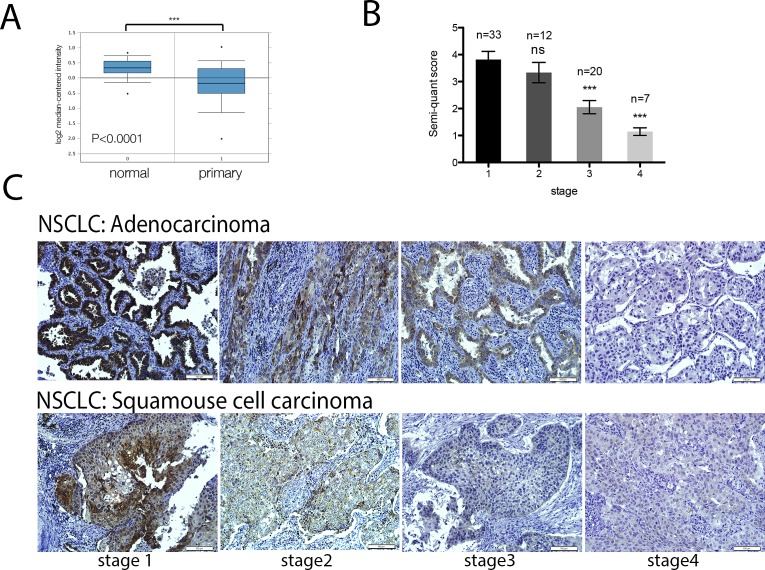
GCIP expression is significantly downregulated in lung cancer tissues A, comparison of GCIP mRNA levels in normal lung and NSCLC tissues (data from the Oncomine database). ^***^ *P*<0.0001 based on Student's t-test analysis. B, GCIP expression in NSCLC lung tissue was 1.86-fold less in stage 3 tumors and 3.34-fold less in stage 4 tumors as compared to stage 1 NSCLC lung tumors. C, prognostic role of GCIP protein levels in NSCLC (adenocarcinoma and squamous cell carcinoma) specimens were examined by immunohistochemical staining (magnification x20). Scale bars, 100 μm.

To investigate the role of GCIP in NSCLC progression, we first assessed its expression in NSCLC gene expression data sets. The Oncomine database revealed that *GCIP* is significantly downregulated in 58 primary NSCLC tissues, compared with adjacent normal tissues (Fig. 1A, *P*<0.0001). We then investigated GCIP expression levels in 72 NSCLC specimens (comprised 68.1% adenocarcinoma and 31.9% squamous cell carcinoma) by Immunohistochemistry. The result revealed significant downregulation of GCIP in both the adenocarcinoma and the squamous cell carcinoma (Fig. 1B). In addition, GCIP expression levels were lower in stage IV tumors as compared to stage I tumor (Fig. 1C). These results suggest that GCIP plays a negative role in tumor progression and may be a valuable biomarker for NSCLC.

### Identification of Id1 as a GCIP-interacting protein

**Figure 2:**
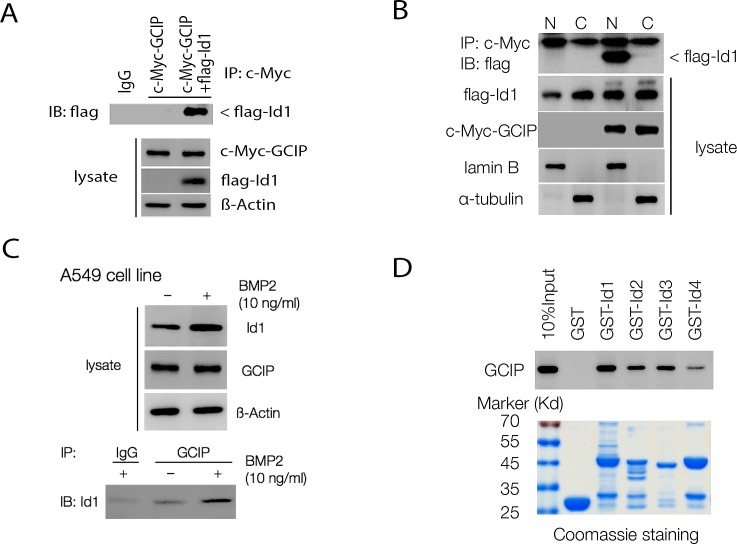
GCIP interacts with Id proteins *in vitro* and *in vivo* A, co-immunoprecipitation assay with an anti-c-Myc antibody and immunoblotting using an anti-Flag antibody shows an interaction between Flag-Id1 and c-Myc-GCIP. B, nuclear localization of the c-Myc-GCIP/Flag-Id1 complex. 293T cells were transfected with vectors encoding GCIP and Id1. Cytoplasmic (C) and nuclear (N) fractions were prepared, and 100 μg of proteins from each fraction were subjected to immunoprecipitation analysis with an anti-c-Myc antibody and immunoblotting using an anti-Flag antibody. C, BMP-2 treatment markedly induces endogenous Id1 expression in A549 cells. Immunoprecipitation assay using an anti–GCIP antibody shows increased interaction of GCIP and Id1 with BMP2 treatment. D, GST-pulldown assays were performed with *in vitro*-translated GCIP in the presence of GST–Id (Id1–4) fusion proteins. GST protein was used as a control. The immunoplexes were subjected to SDS–PAGE and probed with an anti-GCIP antibody.

To gain more insight into GCIP function, we isolated proteins that interact with it using full-length GCIP as the bait in a yeast two-hybrid system. Among the positive clones screened from a human cDNA library, two positive clones had high sequence similarity with Id1. To examine whether GCIP interacts with Id1 in mammalian cells, 293T cells were cotransfected with c-Myc-GCIP and Flag-Id1 expression vectors. As shown in Fig. 2A, Flag-Id1 was specifically coimmunoprecipitated using an anti-Myc antibody followed by Immunoblotting with an anti-Flag antibody. Analysis of 293T cell fractions revealed the c-Myc-GCIP Flag-Id1 complex was present mainly in the nucleus (Fig. 2B). In addition, immunofluorescence analysis indicated that GCIP and Id1 were co-localized in the nucleus of H1299 cells (Fig. S1). Furthermore, the interaction between the endogenous proteins was confirmed in A549 cells (Fig. 2C). Because Id1 expression could be induced by bone morphogenetic protein 2 (BMP2) [31], a markedly enhanced level of endogenous Id1 was coimmunoprecipitated in A549 cells stimulated with BMP2 for 24 h (Fig. 2C), further supporting the physical interaction between GCIP and Id1. To examine whether other Id proteins similarly interact with GCIP, four pBIND vectors containing full-length Id1, Id2, Id3, and Id4 cDNAs were constructed. Mammalian two-hybrid assays indicated that Id1 exhibited the strongest interaction with GCIP followed by Id3; its interaction with Id2 and Id4 was relatively weaker (Supplementary Fig. S2A). Similar results were obtained with GST pull-down assays (Fig. 2D). Using truncated protein constructions, mammalian two-hybrid assays revealed that GCIP requires its HLH domain to associate with Id1 (Supplementary Fig. S2B). Taken together, these results indicated that GCIP more specifically interacts with Id1, compared with the other Id proteins, and this interaction occurs in the nucleus. Furthermore, the HLH domain of GCIP is required for its association with Id1.

### GCIP inhibited and Id1 increased NSCLC proliferation *in vitro*

**Figure 3:**
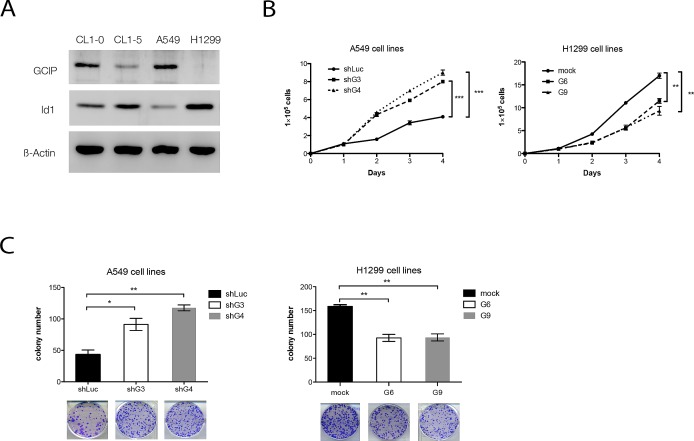
Effects of GCIP over or knockdown on NSCLC cell growth *in vitro* A, endogenous expression of GCIP and Id1 in CL1-0, CL1-5, A549 and H1299, as measured by Western blot analysis. B, cell proliferation rate was determined by cell counting using the trypan blue exclusion assay. C, GCIP expression level–dependent decrease in colony formation. A549 (left) or H1299 (right) cell clones (500 cells/well) were seeded in a 6-well dish. After 2 weeks, the cells were stained with crystal violet and were counted by phase-contrast microscopy. All values are means ± SD from at least three experiments. ^*^P<0.05; ^**^P<0.01; ^***^P<0.001 based on the Student's t-test.

In the less-invasive NSCLS cell lines, CL1-0 and A549. Western blot analysis indicated that relatively high levels of GCIP expression and relatively low levels of Id1expression were detected (Fig. 3A). In contrast, low levels of GCIP expression and high levels of Id1 expression were detected in the highly invasive NSCLC cell lines, CL1-5 and H1299 (Fig. 3A). To explore the functional significance of GCIP-Id1 interaction, two stable H1299 cells with ectopic expression of GCIP (H1299/GCIP clone 6, 9) and two A549 cells stably expressing GCIP shRNA (A549/shGCIP clone 3, 4) were next generated. Western blot analysis confirmed the high expression of GCIP in H1299/GCIP-9 and H1299/GCIP-6 cells (Supplementary Fig. S3A) and reduced GCIP expression level in A549/shGCIP-3 cells as compared to controls (Supplementary Fig. S3B).

Consistent with a previous study [20], both H1299/GCIP-6 and -9 cells exhibited a decrease in cell proliferation (Fig. 3B, right). In contrast, both A549/shGCIP-3 and -4 cells showed a significant increase in cell proliferation, when compared with respective controls (Fig. 3B, left). These results were further confirmed by colony formation assays (Fig. 3C). We also generated stable A549 cells that overexpress Id1 (A549/Id1) and stable H1299 cells that express shId1 (H1299/shId1) (Supplementary Fig. S3C and D). In accordance with a previous study by Yu-Jen Cheng et al. [32], A549/Id1 cells exhibited significantly increased cell proliferation, whereas H1299/shId1 cells displayed significantly decreased cell proliferation, when compared with respective controls (Supplementary Fig. 4A and B).

### GCIP suppresses NSCLC cell tumorigenicity *in vitro* and *in vivo*

**Figure 4:**
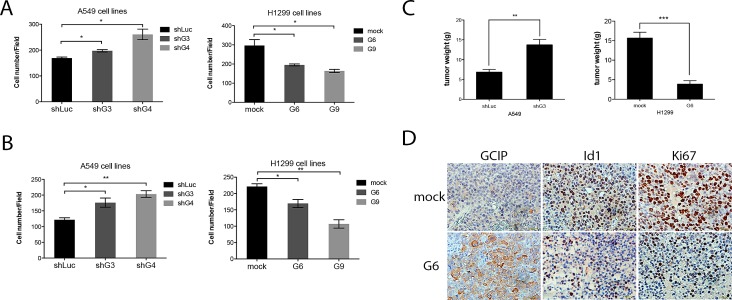
GCIP decreases and Id1 increases the migration and invasion of NSCLC cancer cells *in vitro* and GCIP suppresses tumor growth *in vivo* Effects of GCIP on the migration and invasion of NSCLC cell lines. A, A549 (left) or H1299 (right) stable clones were placed in the upper chamber of a Trans-well and allowed to migrate for 24 h. B, A549 (left) or H1299 (right) stable clones were placed in the upper Matrigel invasion chamber and allowed to migrate for 24 h. Cells that had invaded to the bottom of the membrane were counted manually after staining with crystal violet. C, GCIP-mediated control of *in vivo* tumorigenesis. For each injection, stable A549/shLuc, A549/shGCIP-3, H1299/mock, H1299/GCIP-6 cells were subcutaneously implanted into 4 to 6-week-old female NOD/SCID mice. Five mice were used for each group (points, mean; bars, SEM). D, sections of tumors from mice were analyzed for GCIP, Id1, and Ki67 expression by immunohistochemistry (×40 magnification). ^*,**,***^ *P*≤0.05 based on Student's t-test analysis.

Boyden Chamber assays indicated that H1299/GCIP-9 cells had markedly diminished migration and invasion, whereas A549/shGCIP-4 cells exhibited profoundly increased migration and invasion, when compared with respective controls (Fig. 4A and B). Whereas knockdown of Id1 diminished cell migration and invasion, its overexpression led to enhanced migration and invasion by H1299/shId1 cells and A549/Id1cells, respectively (Supplementary Fig. 5A and B). Consistent with the *in vitro* experiments, A549/shGCIP-4 cells had significantly greater tumor growth, whereas H1299/GCIP-9 cells exhibited reduced tumor growth in NOC/SCID mice (Fig. 4C). Specifically, the average tumor volume in mice bearing A549/shGCIP-4 cells was increased by 50%, whereas mice bearing H1299/GCIP-9 cells had tumor volumes that were decreased by 24%, when compared to respective controls (Fig. 4C). Notably, immunohistochemical staining revealed that the H1299 GCIP-expressing tumors had significantly decreased Ki67-staining relative to control tumors (Fig. 4D). Collectively, our data indicate that GCIP expression suppresses tumorigenicity of NSCLC cells *in vitro* and *in vivo.*

### GCIP suppresses NSCLC progression by interfering with Id1 signaling

**Figure 5:**
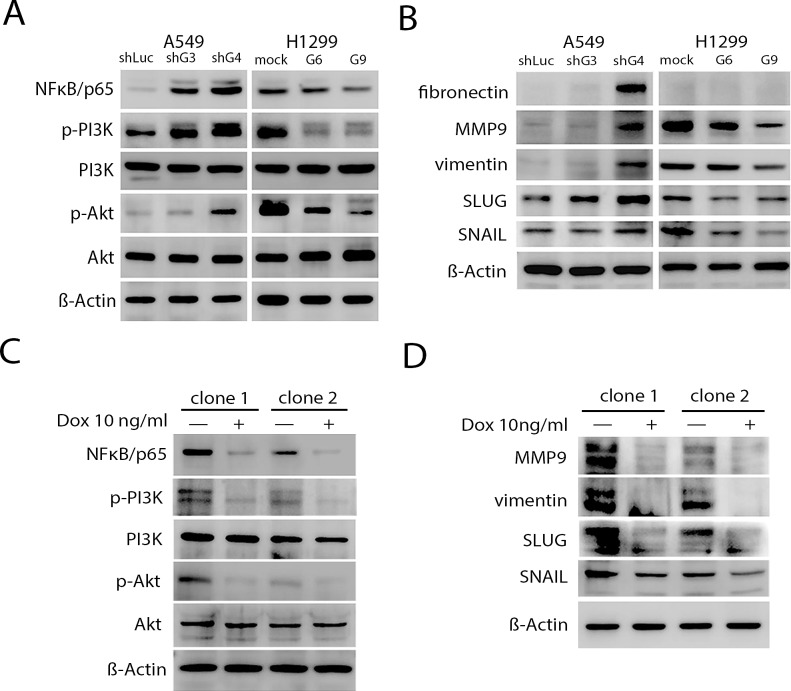
Inhibition of Id-1-induced PI3K/Akt/NFκB signaling- and EMT-associated proteins by GCIP A, The expression of PI3K, phosphorylated PI3K (p-PI3K), Akt, phosphorylated Akt (p-Akt) and NFκB/p65 in A549 and H1299 stable clones. The A549 stable clones had markedly higher expression of p-PI3K, p-Akt and NFκB/p65 than the H1299 stable clones. B, immunoblot analysis showed reduced expression of EMT markers in H1299 stable cells compared with vector control cells and the A549 stable clones. C, Expression of PI3K/Akt/NFκB signaling proteins in doxycycline-induced cells in the presence of Id1. D, MMP9, vimentin, SLUG and SNAIL protein expression was detected by immunoblot assays using specific antibodies. GCIP expression was induced by growing cells in the presence of 10 ng/mL doxycycline for 2 days. Samples were normalized for levels of β-actin.

Previous studies demonstrated that Id1 expression was associated with activation of the Akt-related pathway and correlated with elevated expression of mesenchymal markers in NSCLC cells [16, 32]. Consistent with these studies, we observed a considerable reduction of phosphorylated-PI3K (Tyr467), phosphorylated-Akt (Ser473), NFκB/p65, the EMT inducers or markers (e.g., snail, slug, vimentin and fibronectin), and MMP9 expression levels in H1299/shId1 cells; a significant increase in p-PI3K (Tyr467), p-Akt (Ser473), NFκB/p65, snail, slug, vimentin, fibronectin, and MMP9 expression levels in the A549/Id1 cells was also observed (Supplementary Fig. S6A and B). Furthermore, whereas both H1299/GCIP-6 and -9 cells had significantly decreased p-PI3K (Tyr467), p-Akt (Ser473), NFκB/p65, snail, slug, vimentin, fibronectin, and MMP9 expression levels, both A549/shGCIP-3 and -4 cells displayed significantly increased p-PI3K (Tyr467), p-Akt (Ser473), NFκB/p65, snail, slug, vimentin, fibronectin, and MMP9 expression levels, when compared with their respective controls (Fig. 5A and B).

To determine if GCIP suppression of NSCLC progression may be mediated by inhibiting the Id1 signaling pathway, a stable Tet-On system for doxycycline-dependent expression of GCIP was established in A549/Id1 cells (Supplementary Fig. S6C). Expression of p-PI3K (Tyr467), p-Akt (Ser473), NFκB/p65, snail, slug, vimentin, and MMP9, which was elevated by overexpressing Id1 in A549/Id1/Tet-on-GCIP cells (clone −1 and −2), was profoundly decreased when GCIP was expressed (Fig. 5C and D). Taken together, these data reveal that GCIP suppresses NSCLC progression is, at least in part, mediated by inhibiting Id1 downstream signaling.

### GCIP and Id1 are inversely expressed in NSCLC cell lines and tissues

**Figure 6:**
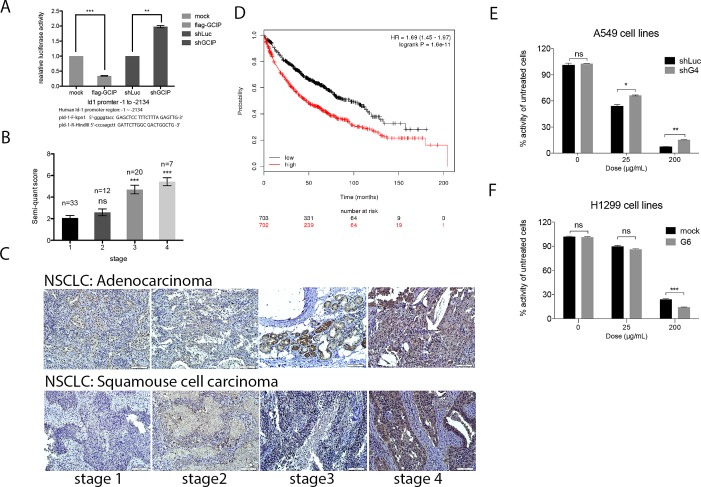
GCIP downregulation of Id1 mRNA expression in NSCLC cells A, A549 cells were triple-transfected with the full-length *Id1* promoter reporter construct, pSV-β-Galactosidase plus Flag-GCIP or shGCIP. Cells were then incubated in 10% FBS DMEM for 24 h. The primers used for the construction of the *Id1* promoter are indicated at the bottom. B, quantification of Id1 expression by IHC analysis of lung cancer specimens. The number (n) of samples for each stage is indicated on the top. C, Id1 expression levels in adenocarcinoma (upper panel) and squamous cell carcinoma (lower panel) of NSCLC specimens by immunohistochemical analysis (magnification x20). Scale bars, 100 μm. Higher Id1 expression levels in NSCLC stages III and IV were observed compared with stage I tissues. Scale bars, 100 μm. D, the data from Kaplan-Meier overall survival analysis shows that Id1 is associated with poor prognosis. ^***^P<0.001 based on Student's t-test. GCIP increased sensitivity to carboplatin in NSCLC cell lines. E, A549/shLuc, A549/shGCIP-4 cells were treated with 0, 25 and 200 μg/mL carboplatin. F, H1299/mock, H1299/GCIP-9 cells were treated with 0, 25 and 200 μg/mL carboplatin. Cell proliferation was measured using an MTT assay (72 h after treatment) and showed that GCIP upregulation significantly sensitized cells to therapy. ^*^P<0.05; ^**^P<0.01; ^***^P<0.001; ns: not significant.

Although Id1 overexpression and silencing did not alter GCIP expression in A549/Id1 and H1299/shId1 cells, respectively (Supplementary Fig. S7A), we observed a marked decrease in Id1 expression in the GCIP-overexpressing (H1299/GCIP-9) cells and a significant increase in Id1 expression in the GCIP-silenced (A549/shGCIP-3 and -4) cells (Supplementary Fig. S7B). Co-transfection of an *Id1* promoter reporter construct (from −1 to −2134) [13] with a GCIP expression plasmid, a GCIP-shRNA plasmid, or control vector, revealed that ectopic expression of GCIP led to markedly decreased *Id1* promoter reporter activity; expression of shGCIP led to upregulated *Id1* promoter reporter activity (Fig. 6A). Thus, GCIP expression downregulates Id1 expression by inhibiting its transcription, and silencing GCIP upregulates Id1 expression in NSCLC cell lines.

The inverse correlation between GCIP and Id1 expression was also observed in NSCLC specimens. As shown in Fig. 6B, Id1 was weakly expressed in early-stage NSCLC specimens; however, its expression increased with malignancy. Furthermore, Id1 expression analysis was also conducted using the same samples employed for GCIP IHC analysis in Fig. 1B. Low levels of GCIP expression significantly correlated with high levels of Id1 expression in the metastatic tumors (Fig. 6C). This inverse expression pattern between GCIP and Id1 was observed in the NSCLC tissues from 72 human patients; there was a statistically significant inverse correlation between GCIP and Id1 expression (r=-0.2834, *p*< 0.05). A previous study reported that higher Id1 levels were associated with an overall survival in adenocarcinoma but not in squamous cell carcinoma from 346 NSCLC patients[15], however survival analysis using the Kaplan-Meier plotter database (www.kmplot.com, 2014 version) with a log-rank test revealed that increased Id1 expression correlated with poor prognosis in both the adenocarcinoma and squamous cell carcinoma from 1405 NSCLC patients (Fig. 6D and Supplementary Fig. S7C). Nevertheless no information of GCIP expression can be seen in this database. Taken together, GCIP inhibits the expression of Id1, and GCIP and Id1 are inversely expressed in NSCLC cell lines and tissue specimens, suggesting that GCIP suppresses NSCLC progression by downregulating the expression and function of Id1.

### Effects of GCIP on sensitivity of NSCLC cells to chemotherapy

Because increased Id1 expression was correlated with chemoresistance of NSCLC cells [15], we examined whether overexpression of GCIP could reverse this chemoresistance. GCIP-overexpressing H1299 cells (H1299/GCIP-9), GCIP-silenced A549 cells (A549/shGCIP-4), or their respective control cells were treated with 0, 25 and 200 μg/mL carboplatin for 72 h. As expected, H1299/GCIP-9 cells treated with 200 μg/mL carboplatin exhibited a significant reduction in cell growth compared to the H1299 cells treated with the same dose (*p*<0.05; Figure 6F). On the contrary, A549/shGCIP-4 cells exhibited a significantly increased the cell viability when treated with 25 or 200 μg/ml carboplatin compared to the A549 cells treated with the same dose (*p*<0.05; Fig. 6E). Similar results were also obtained from these cells treated with docetaxel, which has been approved as a first- and second-line treatment for advanced NSCLC (Supplementary Fig. S7D and E). Thus, depletion of GCIP promoted resistance, while expression of GCIP potentiated the cytotoxic effect of anticancer drugs, suggesting that GCIP expression increased susceptibility to chemotherapeutic agents by inhibiting Id1-induced chemoresistance. Furthermore, depletion of GCIP, which was observed in most NSCLC specimens especially in patients with invasive NSCLC, might reduce chemosensitivity in NSCLC patients.

## DISCUSSION

Growing evidence indicates that the GCIP is a novel tumor suppressor in breast, colon and liver cancers [22, 27-29]. However, the precise mechanisms underlying its anti-tumor properties have not been fully elucidated. It is generally thought that GCIP may inhibit the protein expression and function of cyclin D1, and thereby, inhibiting cell proliferation and tumorigenesis [18, 20, 22-24, 27, 28]. Consistent with these previous studies, in this study, we also found that both cyclin D1 expression and phosphorylation of Rb at Ser780 (RbS780) were enhanced in GCIP-silenced A549 cells; both cyclin D1 and RbS780 were decreased in GCIP-overexpressing H1299 cells (data not shown). Furthermore, our data presented here also demonstrated that GCIP interacts with and represses Id in NSCLC cell lines. Since cyclin D1 has been implication in several cell types as a target of Id1 [33, 34] and is a key modulator proliferation, it is reasonable to speculate that attenuation of GCIP would lead to upregulation of Id1, which may also contribute to enhanced cyclin D1 and RbS780 levels, subsequently leading to the increased proliferation.

In addition to inducing cell proliferation, Id1 may also induce EMT, inhibit apoptosis and promote migration, invasion, metastasis and drug resistance in several types of cancer [35, 36]. It is an upstream regulator of the PI3K/Akt/NFκB pathway in esophageal, head and neck, ovarian, gastric and lung cancer cells [32, 37, 38], and the expression of vimentin and fibronectin is regulated by Id1 at transcriptional level in NSCLC [16]. In addition, Id1 induces snail1expression in kidney epithelial cells [39] and positively regulates MMP-9 expression in breast and lung cancer cell lines [40, 41]. Consistent with these previous studies, Id1-induced expression and/or activation of the aforementioned proteins was inhibited in GCIP-overexpressing H1299 cells, and was enhanced in GCIP-silenced A549 cells. Furthermore, GCIP downregulated these proteins through inhibition of Id1, which was confirmed in a conditionally induced stable A549 cell line. Significantly higher Id1 protein expression was previously found in 346, 61[15, 41] and 1405 NSCLC specimens (www.kmplot.com, 2014 version), respectively, and 72 NSCLC specimens in this study. As compared to Id3, more frequent expression of Id1 was observed in NSCLC specimens [42]. Furthermore, among the Id proteins, GCIP exhibited the strongest interaction with Id1, inhibiting its expression. Given the role of the PI3K/Akt/NFκB pathway in apoptosis resistance [38, 43], chemoresistance [44, 45] as well as the regulation of cellular invasion and metastasis [46, 47] as well as the importance of both snail and Slug as critical regulators of EMT, which is important for tumor invasion and metastasis, and resistance to several antitumor-drugs [48, 49] combined with the role of MMP9 as an important mediator of malignant invasiveness and metastasis [40], it is tempting to speculate that GCIP-mediated downregulation of Id1 and its downstream effectors could be a key mechanism by which GCIP can potently suppress NSCLC tumor progression.

Id1 is recognized as an inhibitor of differentiation and essential for embryonic development [6]. Consistent with dedifferentiation of adult tissues being intricately connected to oncogenesis, the evidence for an important role of Id1 in cancer development and progression seems to be growing [35]. Alternatively, although GCIP is also recognized as a dnHLH protein and structurally related to the mouse Id1-like molecule, Maid, it is widely expressed in differentiated cells in adults and has been demonstrated to be a tumor suppressor in breast, colon and liver cancers in contrast to the extremely low expression of Id1 in adult tissues. Induction of Id1 expression in response to extracellular signaling molecules, including bone morphogenetic proteins (BMPs), has also been reported [31, 50, 51]. In addition, previous studies have also demonstrated that Id1 is required to maintain self-renewal capacity in various stem cells [13, 52], and is required for tumor initiating function, both in the context of primary tumor formation and during metastatic colonization of the lung microenvironment [53]. In this study, we demonstrated that GCIP transcriptionally inhibited *Id1* expression and exerted antagonistic effects on Id1-driven tumor progression of NSCLC. On the basis of these findings, and the fact that GCIP is expressed mainly in terminally differentiated tissues in adults where Id1 levels are extremely low, we propose that GCIP expression in both the non-transformed lung cells and the differentiated NSCLC cells serves to restrict the levels of Id1. However, in the presence of extracellular signaling molecules that can induce Id1 but not GCIP expression in NSCLC cell lines (e.g., BMP2, Fig. 1C), upregulation of Id1 may overcome the tumor suppressive functions of GCIP. In addition, loss of GCIP through currently unidentified mechanisms during tumor development could also contribute to enhanced Id1 levels, consequently promoting tumor progression of NSCLC. The regulation of GCIP and the mechanisms that GCIP transcriptionally regulates Id1 are currently being studied.

Our present study also showed that GCIP expression level negatively correlated with NSCLC disease stage, and that GCIP and Id1 are inversely expressed in NSCLC cell lines and tissues. In addition, we also demonstrated that GCIP downregulated Id1 to render NSCLC cells more susceptibility to carboplatin and docetaxel, two commonly used anticancer drugs for NSCLC. Thus, GCIP may also be a useful prognostic marker for NSCLC in addition to Id1. Furthermore, our data also imply that robust induction of GCIP may not only improve chemosensitivity but also be an effective therapeutic approach in NSCLC.

In summary, GCIP is a tumor suppressor that interacts with Id1 in NSCLC. Upregulation of GCIP suppresses Id1-mediated downstream signaling. Clinical and mechanistic evidence supports that GCIP exerts its tumor-suppressive function in NSCLC through suppression of Id1-triggered oncogenic properties.

## MATERIALS AND METHODS

### Cell lines

293T cells and the lung cancer cell lines, A549, H1299, CL1-0 and CL1-5, were cultured in complete medium consisting of Dulbecco's Modified Eagle's medium (DMEM, Gibco, Grand Island, NY, USA) and 10% fetal bovine serum (FBS, Invitrogen, Carlsbad, CA, USA). All cells were incubated in 10-cm tissue culture dishs (BD Falcon, San Jose, CA, USA) at 37°C and 5% CO_2_, and were subcultured every 3–4 days. P.C. Yang kindly provided the lung cancer cell lines, CL1-0 and CL1-5. The lung cancer cell lines, A549 and H1299, were purchased from Bioresource Collection and Research Center (BCRC), Hsinchu, Taiwan.

### Immunoprecipitation

Cells that were transfected with plasmids as described in Supplementary Methods were washed with cold PBS and lysed in NP-40 buffer (NaCl, 150 mM, NP-40, 1.0%, Tris-Cl 50 mM, pH 8.0). A detailed procedure for the immunoprecipitation assay was described in the Supplementary Data.

### GST pull-down assay

BL-21 bacteria were transformed with pGEX-5X-3 (GST alone) or each GST fusion construct a described in Supplementary Methods. A detailed procedure for the GST pull-down assay was described in the Supplementary Data.

### Id1 promoter assays

Luciferase and galactosidase assay systems (Promega, Madison, WI, USA) were used for promoter analysis. A detailed procedure for the Id1 promoter analysis with GCIP was described in the Supplementary Data.

### Patient clinical specimens and immunohistochemistry

NSCLC tissue samples were collected from 72 consecutive patients, who underwent resection at the National Cheng Kung University Hospital. All patients gave informed consent for retention and analysis of their tissue for research. Unstained slides were deparaffinized in xylene and rehydrated sequentially in ethanol. Immunohistochemistry analysis of GCIP was performed as previously described[29] and Id1 was described in the Supplementary Data.

### Proliferation, migration, invasion and clonogenic assays

Cell proliferation was measured by trypan-blue staining. Briefly, cells were plated at a concentration of 1 × 10^5^ cells in 10-cm dishes. Every 24 h, cells were trypsinized and counted. To evaluate migration or invasion, 1–5 × 10^4^ cells were cultured in serum-free DMEM in an upper compartment of a transwell (BD Biosciences) or with a matrigel-coated transwell (BD Biosciences) using 24-well plates for 24 to 72 h. The wiped membrane was fixed and stained with 0.5% crystal violet. The percentages of migrating cells were calculated in three independent representative fields. Colony formation assays were conducted by plating 500 cells/well into 100-mm culture dishes in triplicate. After a 12-day incubation at 37°C and 5% CO_2_, cells were fixed with formaldehyde and stained with 2% crystal violet. The number of colonies was then counted, and the surviving fraction of treated cells was normalized to the surviving fraction of the corresponding controls.

### Xenograft

To examine the effect of GCIP expression in tumor formation, 1 × 10^6^ H1299/mock, H1299/G6, A549/shLuc or A549/shG3 cells were implanted subcutaneously into 5-week-old female BALB/c nude mice (n=5), and the tumor growth was monitored using calipers every 3 to 4 days. Animals were sacrificed 30 or 35 days after inoculation, and tumors were isolated and fixed in 10% natural-buffer formalin (Merck, Whitehouse Station, NJ, USA) for further immunohistochemical staining was performed as previously described [29].

### Expression dataset from microarray analysis

The Oncomine databases were queried to identify the prognostic role of GCIP components in NSCLC. The Oncomine Cancer Microarray database from Hou et al. [30] was employed to compare GCIP gene expression in normal lung and primary in NSCLC tumor.

### Cell viability analysis by MTT assay

A549/shLuc, A549/shG3, H1299/mock and H1299/G6 cells were plated in 96-well plates (2 ×10^6^ cells/mL) in triplicate. Cells were treated with 0, 25, and 200 μg/mL carboplatin or 0, 25 μg/mL docetaxel. After 3 days, cell proliferation was measured using MTT (Sigma, St. Louis, MO, USA) assays according to the manufacturer's protocol. Absorbance was measured at 490 nm.

### Scoring and statistical analysis

Staining intensities (1 = +, 2 = ++ and 3 = +++), fraction of positive cells (0=0%, 1=<5%, 2=5–50% and 3=>50% of tumor cells) and the histological grade (G1 = well, G2 = moderate and G3 = poor) were scored by an experienced pathologist (MG), who was blinded to the patient's clinical details. The semi-quantitative H-score, defined as the staining intensity × fraction of positive cells (range, 0–9), was determined and used for statistical analysis. Pearson rank correlation (r) was used to assess correlation between *GCIP* and *Id1* genes. Unless otherwise specified, all experiments were performed in triplicate and were repeated at least twice. Data were expressed as mean values ±SE or SD and were analyzed using GraphPad Prism software. The level of significance was set at *P* < 0.05.

## SUPPLEMENTARY MATERIALS, METHODS AND FIGURES


