# Supplementary material for: GCIP functions as a tumor suppressor in non-small cell lung cancer by suppressing Id1-mediated tumor promotion
Source: Oncotarget. 2014 Jun 7;5(13):5017–28. doi: 10.18632/oncotarget.2075 (PMC4148118; doi:10.18632/oncotarget.2075)
Supplement: Supplementary file 1 [file oncotarget-05-5017-s001.pdf]

GCIP functions as a tumor suppressor in non-small cell lung cancer by suppressing Id1-mediated tumor promotion

Supplementary materials and methods

Plasmids and stable transfection

All constructs were generated by PCR using primers designed from the coding regions of the relevant human cDNAs. Full-length GCIP was amplified by PCR and inserted into pcDNA3.1B (Invitrogen, Carlsbad, CA, USA), pACT (Promega, Madison, WI, USA), pT7CFE1-Chis (Thermo Scientific, Dharmacon Division) or pTRE3G-ZsGreen (Clontech, Palo Alto, CA, USA), respectively. Full-length Id1 cDNA was amplified by PCR and inserted into p3X-Flag (Sigma, St. Louis, MO, USA). The truncated Id1 fragment (coding 53–105 amino acids) or GCIP fragment (coding 83–324 amino acids) was cloned into the pACT or pBIND (Promega, Madison, WI, USA), respectively. Full-length Id1, Id2, Id3 and Id4 cDNA was amplified by PCR and inserted into pBIND or pGEX-5X-3, respectively. For infection, full-length GCIP was amplified and subcloned into pLKO-AS3w vector (Academia Sinica, Taipei, Taiwan). GCIP shRNA-expressing lentiviral plasmid 1 and 2 were constructed by cloning synthesized shRNA into pLKO.1-puro vector, respectively. GCIP-targeting sequences were 5′-CCACAATCATGAGGATGAT-3′ and 5′-GACTCAATGAGGCAGCTGT-3′. The pLKO.1-lentivirus constructs carrying small hairpin RNA (shRNA) against Id1, were obtained from the National RNAi Core Facility, Academia Sinica, Taipei, Taiwan. The human Id1promoter (-1 to -2134) fragment reporter construct was generated by PCR amplification of human genomic DNA, followed by insertion into the multiple cloning site of the pGL3 vector (Promega). All the constructions were confirmed by restriction enzyme mapping and

DNA sequencing. Stable cell line overexpressing Id1 was selected with changes of fresh medium containing G418 (600 μg/mL).

Lentivirus production and infection

Lentiviral vector carrying shRNA construct against GCIP and Id1 or full-length GCIP insert was prepared using a three-plasmid transfection method. Briefly, the pMD.G containing vesicular stomatitis virus glycoprotein, and pCMV-Δ 8.91carrying HIV-based packaging plasmid were co-transfected with lentiviral vector (control) or with the shRNA-, or GCIP-expressing lentiviral vector into 293T cells. The medium was changed to fresh DMEM containing 10 mg/ml BSA 24 hours post-transfection. The supernatant containing lentivirus was collected at 48 hour and 72 hour post-transfection, respectively. To infect A549 or H1299 cells, lentiviral supernatant was added directly to cells in the presence of 8 μg/ml polybrene, respectively. Following infection, the cells were selected using 5 μg/ml puromycin. A549/shG3 cells GCIP-targeting sequences were 5′-CCACAATCATGAGGATGAT-3′ and shG4 cells GCIP-targeting sequences were 5′-GACTCAATGAGGCAGCTGT-3′. H1299/G6 and G9 were from single clones using one plasmid.

Western blotting and antibodies

Cells were washed with cold phosphate-buffered saline (PBS) and lysed in RIPA lysis buffer (Millipore, Billerica, MA, USA). Equal amounts of protein (50 μg) were separated by 10–12% sodium dodecyl sulfate– polyacrylamide gel (SDS–PAGE) and transferred to PVDF membrane (Millipore). The membranes were blocked with TBST buffer containing 5% non-fat milk, and incubated with indicated primary antibody. The membranes were then incubated with HRP-conjugated secondary antibodies and

were visualized using the ECL chemiluminescent method (Millipore). The primary antibodies the following antibodies: GCIP, PI3K, AkT, fibronectin and vimentin (BD Biosciences Pharmingen, San Diego, CA); NFκB/p65, p-PI3K (Tyr467), p-Akt (Ser473), α-tubulin (Genetex Inc., CA, USA); Myc tag, β-Actin, Ki67 (Millipore); SLUG, SANIL (Cell Signaling Technology, Beverly, MA); Flag (Sigma-Aldrich); Lamin B (Zymed Laboratories Inc., South San Francisco, California, USA); MMP9 (Abcam Inc., Cambridge, MA, USA); Id1 (Santa Cruz Biotechnology, Santa Cruz, CA, USA). The HRP-conjugated goat anti-mouse and anti-rabbit secondary antibodies were purchased from Chemicon (Temecula, CA, USA).

Immunohistochemistry

Formalin-fixed paraffin-embedded tumor tissue sections (obtained from the National Cheng Kung University Hospital) were used to determine Id-1 protein expression in 72 NSCLC tissue samples. Slides were dewaxed, rehydrated, and placed in a container containing 1 liter of 0.01 M citrate buffer (pH 6.0); they were then microwaved at 700 W for 20 min, allowed to remain in the hot citrate buffer for 15 min, and cooled down in running cold water. The slides were washed in deionized water and incubated in 10% nonfat dry milk for 30 min at room temperature, washed in TBS, and incubated with 1 μg/ml of anti Id-1 antibody overnight at 4°C. Control slides were incubated with rabbit immunoglobulin. The slides were washed in TBS and incubated with biotinylated swine anti-rabbit fragments (1: 400) for 30 min. After washing in TBS, endogenous peroxidase was blocked with 0.3% hydrogen peroxide and 0.1% sodium azide for 10 min. The slides were washed in TBS and incubated with 1:500 streptavidin-horseradish peroxidase for 30 min. After washing in TBS, peroxidase was visualized by incubating in 0.5 mg/ml diaminobenzidine-4-HCl and

0.03% hydrogen peroxide in TBS for 3 min. The slides were washed in TBS and water before mounting.

Immunoprecipitation

Cells that were transfected with plasmids as described above were washed with cold PBS and lysed in NP-40 buffer (NaCl, 150 mM, NP-40, 1.0%, Tris-Cl 50 mM, pH 8.0). Then 500 μg of cell lysates were incubated with 2–5 μg of the indicated antibodies for 10–14 h at 4°C. Fifty microliters of protein A- agarose (GE Healthcare Life Sciences, Piscataway, NJ, USA) was added and incubated; the immune-complexes were resolved by SDS–PAGE followed by western blotting.

GST pull-down assay

BL-21 bacteria were transformed with pGEX-5X-3 (GST alone) or each GST fusion construct as described above and cultured in an incubator at 37 °C; 1 mM IPTG was added to the culture to induce expression of GST or each GST-fusion protein. Bacteria were pelleted and lysed in buffer (40 mM Tris–HCl (pH 7.5), 150 mM NaCl, 1 mM dithiothreitol, 1 mM EDTA, 0.5% NP40, 10% glycerol, 0.4 mM phenylmethylsulfonyl fluoride, 2 Ug/ml leupeptin, and 2 Ug/ml aprotinin) along with lysozyme (5 mg/l) and a bacterial protease inhibitor cocktail (Sigma-Aldrich). Suspensions were vortexed to resuspend the pellet, incubated on ice for 30 min, and sonicated for 5 min. The insoluble fraction was removed by centrifugation, and the supernatants incubated with glutathione-Sepharose beads (GE Healthcare Life Sciences) for 30 min at room temperature. For the pull-down assays, 30 μl of the 50% GST or each GST-fusion protein bead slurry were incubated with recombinant human GCIP proteins from in vitro transcription-translation reactions (Thermo scientific). To

detect protein that bound specifically, the beads were washed six times with lysis buffer, boiled in SDS-PAGE samples buffer, and subjected to SDS-PAGE and analyzed by immunoblotting.

Immunofluorescence

H1299/mock, H1299/GCIP clone 6 cells were fixed with 3.7% formaldehyde in PBS buffer, and permeabilized with 0.2% Triton X-100, and blocked in PBS containing 5% bovine serum albumin. Primary antibodies used were rabbit anti-Id1 (1:200) and mouse anti-GCIP (1:200). The secondary antibodies used were anti-rabbit Alexa 594 (1:500) and anti- mouse Alexa 488 (1:500) (Molecular Probes, Carlsbad, CA, USA). 4', 6'-Diamino-2-phenylindole (DAPI, Molecular Probes) was used to stain the nuclei. Confocal scanning analysis of the cells was done with EZ-C1 confocal imaging system (Nikon).

Mammalian two-hybrid assay

HEK 293T cells were co-transfected with 2 μg of pG5-luc, pSV-β-galactosidase, and each pBIND fusion construct as described above. In the case of a positive control experiment, we transfected cells with 2 μg of pACT-GCIP, which contains the VP16 activation domain, and pBIND-P0 control vector of CheckMateTM mammalian two-hybrid system (Promega) instead of pBIND vector. At 24 h after transfection, cells were washed three times with PBS and scraped in 400 μl of reporter lysis buffer (Promega). The cells were subjected to a single freeze-thaw. After vortexing the cells, the lysates were centrifuged at 10,000 × g for 1 min at 4 °C, and the supernatant was assayed directly or stored at −70 °C. The β-galactosidase activity was assayed using the β-galactosidase assay system with reporter lysis buffer (Promega). Luciferase

activity in 10 μl of the cell extract was measured by an automated luminometer (Wallac- Berthold, Tokyo). Luciferase activity was normalized by β-galactosidase activity, and the data thus normalized by β-galactosidase activity are presented. Each pBIND construct was transfected into three separate dishes, and the results were confirmed in three independent experiments.

Generation of stable doxycycline inducible GCIP clones in A549/Id1 cell

A549/Id1 stable Cell at about 70% confluences was transfected with 2 μg pCMV-Tet3G vector (Clontech) using LipofectAMINE Plus Reagent (Invitrogen) according to the manufacturer's protocol. After transfection, cells were plated in four 10-cm-diameter cell culture dishes. Replace medium with fresh complete medium plus G418 (150 μg/ml) every four days. Appearance of G418 resistant clones was monitored using an inverted microscope. These clones were isolated using cloning cylinders, transferred to individual wells, and screened for screened for expression of transactivator using the Promega Luciferase Assay System (Promega) after transient transfections with pTRE3G-Luc plasmid.Experiments were performed in six-well plates. Then, clones with high expression were transfected with a linear hygromycin plasmid and pTRE3G-GCIP in which wild-type GCIP cDNA was incorporated downstream of Tet-regulated P_{TRE3G}. Induction of GCIP was accomplished by addition of doxycycline. Two out of 24 hygromycin–resistant clones displayed tightly regulated induction of GCIP and were selected for further use.

Id1 promoter assays

Luciferase and β-galactosidase activities were detected in transfected A549 cells using the luciferase (Promega) and galactosidase (Promega) enzyme assay systems.

Briefly, Flag-GCIP or GCIP shRNA-expressing plasmid with Id1pGL3-luc reporter and pSV-ß-Galactosidase as an internal control plasmids at a ratio of 3:5:1 in the presence of Turbofect (Thermo scientific) in 10% FBS DMEM. Transfected cells were harvested 48 h after transfection, and luciferase and galactosidase enzyme activities were assayed according to the manufacturer's instructions. Luciferase activity values were normalized to the β-galactosidase activity values.

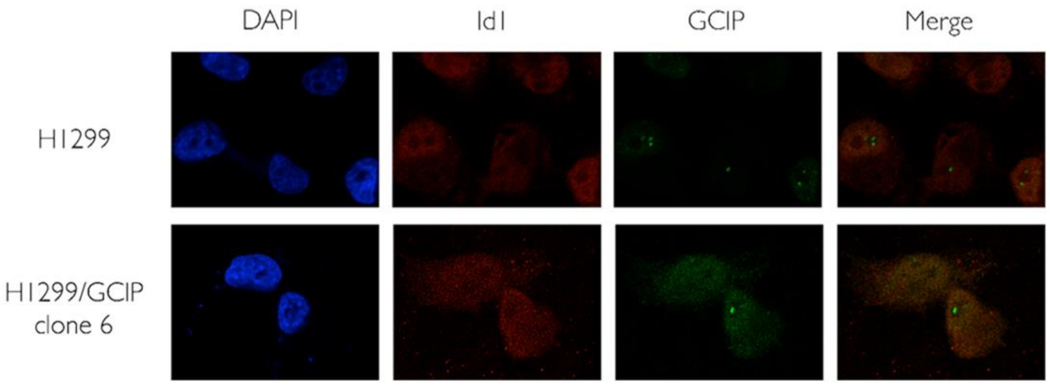

Supplementary Figure 1: H1299/GCIP stable clone 6 cells were immunostained using GCIP (green) and Id1 (red) antibodies and DAPI (blue). GCIP and indigenous Id1 is co-localizaton (yellow) in nuclear.

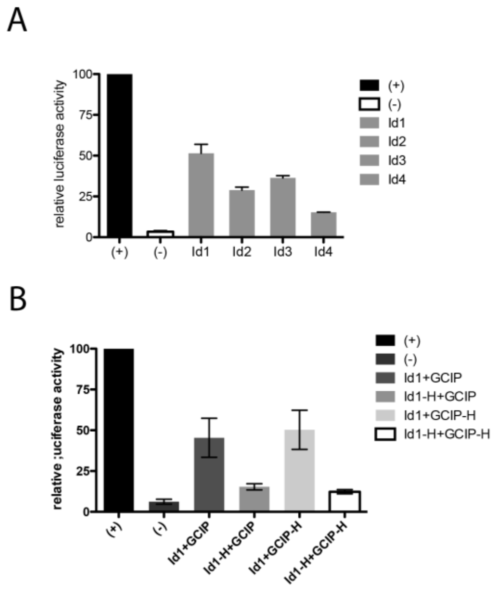

Supplementary Figure 2: Two-hybrid screening identifies Id1 as a GCIP-interacting protein. A, pACT-GCIP and pBIND-Id (1-4) were cotransfected into 293T cells along with pG5*luc* vector to generate fusion proteins. B, HLH region of GCIP (447-570) or Id1 (201-324) were fused to the pACT or pBIND, respectively. Mammalian two-hybrid assays were carried out to test the interaction between GCIP and Id1 functional regions in mammalian cells. The relative luciferase activity levels (normalized with β-Galactosidase system) for pACT-GCIP and pBIND-P0 vectors transfections were arbitrarily assigned a value of 100%. All experiments were performed in triplicate and were repeated at least three times, and the results are expressed as mean ± SEM.

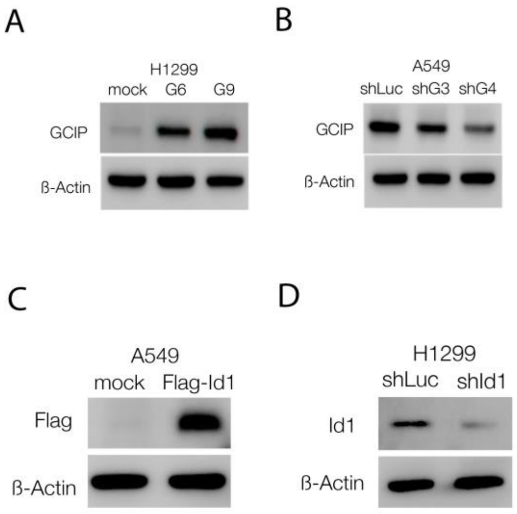

Supplementary Figure 3: NSCLC cell lines A549 and H1299, which were used to generate stable GCIP-expressing and GCIP knockdown clones. A, immunoblotting result showing the expression of GCIP in two GCIP-overexpressing clones (G6 and G9) for H1299 cells. B, immunoblotting result showing the expression of GCIP in two GCIP-knockdowned clones (shG3 and shG4) for A549 cells. C, Western blot of Id1 protein level in clones Flag-Id1, by comparison to clones transfected with the empty p3xFLAG-CMV-26 expression vector. D, Western blot of Id1 protein level in clones shId1, by comparison to clones transfected with the pSilencer 2.1-U6 hygro luciferase. β-Actin was included as internal loading control.

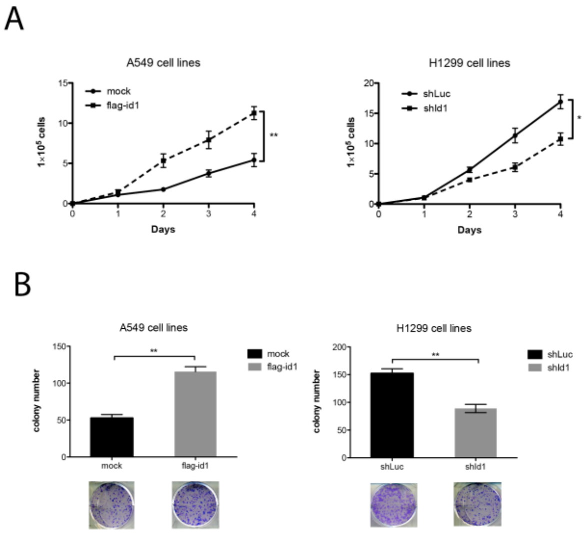

Supplementary Figure 4: Id1 expression significantly enhances NSCLC cells proliferation. A, proliferation of A549 (left) and H1299 (right) stable cells were examined in expression or knockdown of Id1 by counting cell numbers 2, 3 and 4 days after seeding. B, the effect of ectopic expression or knockdown of Id1 on tumor cell growth was investigated by monolayer colony formation assayin A549 (left) or H1299 (right) stable cells. All values are means ± SD from at least three experiments. *, P < 0.05; **, P<0.01 based on Student's t-test analysis.

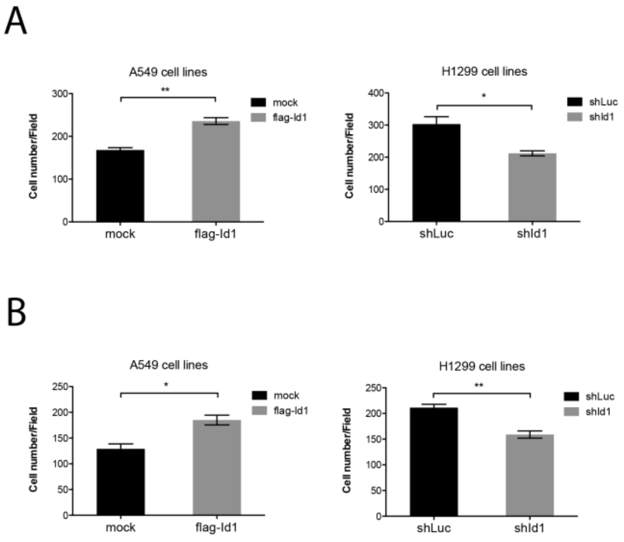

Supplementary Figure 5: Effects of Id1 on migration and invasion of A549 and H1299 stable cell lines. A, overexpression Id1 increase A549 cell migration (left) and Id1 knockdown impairs H1299 cell migration (right). Invasion curves were determined for control and Id1 overexpressed or knockdown cells by counting the cell numbers. B, the invasiveness of A549/mock, A549/Id1 (left), H1299/shLuc and H1299/shId1 (right) cells was compared using Matrigel-coated membranes. All values are means ± SD from at least three experiments. *, $P < 0.05$; **, $P<0.01$ based on Student’s t-test analysis.

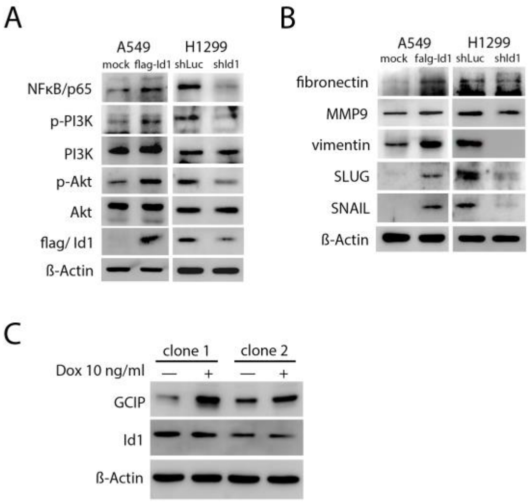

Supplementary Figure 6: A, effects of Id-1 modulation on PI3K/Akt/NF□B signaling pathway as detected by Western blot. B, Western blot analysis of EMT markers in A549/mock, A549/Id1, H1299/shLuc and H1299/shId1 cells. C, Inducible GCIP expression in a stable Tet-On A549/Id1 cell line (A549/Id1/Tet-on-GCIP clone -1 and -2). GCIP induced by doxycycline (Dox) 10 ng/ml concentrations were evaluated by immunoblot assay with GCIP-specific antiserum. β-Actin was included as internal loading control.

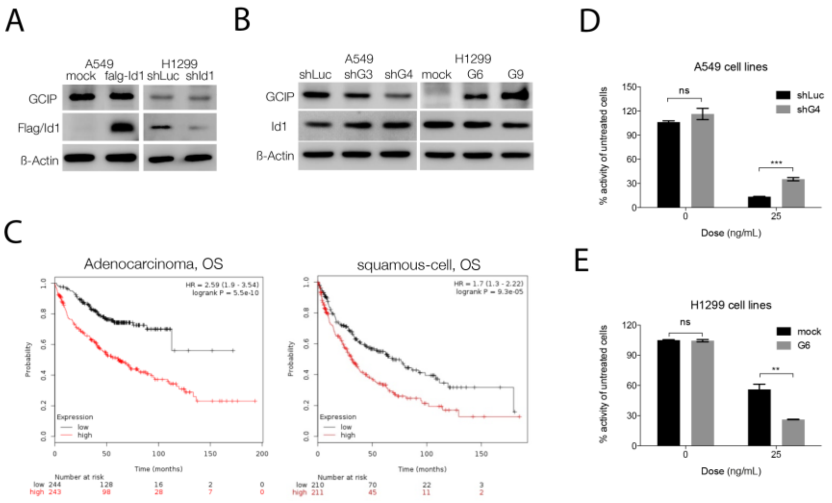

Supplementary Figure 7: A, Cell extracts derived from A549/mock, A549/Flag-Id1, H1299/shLuc and H1299/shId1 cells were analysed for expression of GCIP and Id1 by Western blot. B, Western blotting analyses of GCIP and Id1 in the indicated cell lines; □-Actin was used as loading control. C, the data from Kaplan-Meier overall survival analysis shows that Id1 is associated with poor prognosis in adenocarcinoma (left) and squamous-cell lung cancer (right). $^{***}P<0.001$ based on Student's t-test. GCIP increased sensitivity to carboplatin in NSCLC cell lines. D, A549/shLuc, A549/shGCIP-4 cells were treated with 0 and 25 ng/ml carboplatin. E, H1299/mock, H1299/GCIP-9 cells were treated with 0 and 25 ng/ml carboplatin. Cell proliferation was measured by using an MTT assay (72 h after treatment) and showed that GCIP

upregulation significantly sensitized cells to therapy (**$p<0.01$, ***$p<0.001$; ns: not significant).
